# Effect of Creatine Supplementation on Functional Capacity and Muscle Oxygen Saturation in Patients with Symptomatic Peripheral Arterial Disease: A Pilot Study of a Randomized, Double-Blind Placebo-Controlled Clinical Trial

**DOI:** 10.3390/nu13010149

**Published:** 2021-01-05

**Authors:** Wagner Jorge Ribeiro Domingues, Raphael Mendes Ritti-Dias, Gabriel Grizzo Cucato, Nelson Wolosker, Antônio Eduardo Zerati, Pedro Puech-Leão, Daniel Boari Coelho, Pollyana Mayara Nunhes, André Alberto Moliterno, Ademar Avelar

**Affiliations:** 1 Institute of Social Sciences Education and Zootechnics, Federal University of Amazonas, Parintins 69152-240, Brazil; 2 Postgraduate Program in Rehabilitation Sciences, Nove de Julho University, Sao Paulo 03155-000, Brazil; raphaelritti@gmail.com; 3 Department of Sport, Exercise and Rehabilitation, Northumbria University, Newcastle upon Tyne NE1 8PP, UK; gacucato@gmail.com; 4 Hospital Israelita Albert Einstein, Sao Paulo 05652-900, Brazil; nwolosker@yahoo.com.br; 5 Cancer Institute of Sao Paulo, Sao Paulo 01246-000, Brazil; azerati@uol.com.br; 6 Faculty of Medicine Sao Paulo University, Sao Paulo 04021-001, Brazil; pedro@puech.com.br; 7 Center for Engineering, Modeling and Applied Social Sciences (CECS), Federal University of ABC, São Bernardo do Campo 09606-070, Brazil; danielboari@gmail.com; 8 Department of Physical Education, State University of Maringa, Maringa 87020-900, Brazil; polly_nunhes@hotmail.com (P.M.N.); andremoliterno@hotmail.com (A.A.M.); ademaravelar@yahoo.com.br (A.A.)

**Keywords:** intermittent claudication, mobility limitation, dietary supplements

## Abstract

The aim of the study was to verify the effects of creatine (Cr) supplementation on functional capacity (walking capacity; primary outcome) and calf muscle oxygen saturation (StO_2_) (secondary outcome) in symptomatic peripheral arterial disease (PAD) patients. Twenty-nine patients, of both sexes, were randomized (1:1) in a double-blind manner for administration of placebo (PLA, *n* = 15) or creatine monohydrate (Cr, *n* = 14). The supplementation protocol consisted of 20 g/day for 1 week divided into four equal doses (loading phase), followed by single daily doses of 5 g in the subsequent 7 weeks (maintenance phase). Functional capacity (total walking distance) was assessed by the 6 min walk test, and calf muscle StO_2_ was assessed through near infrared spectroscopy. The measurements were collected before and after loading and after the maintenance phase. The level of significance was *p* < 0.05. No significant differences were found for function capacity (total walking distance (PLA: pre 389 ± 123 m vs. post loading 413 ± 131 m vs. post maintenance 382 ± 99 m; Cr: pre 373 ± 149 m vs. post loading 390 ± 115 m vs. post maintenance 369 ± 115 m, *p* = 0.170) and the calf muscle StO_2_ parameters (*p* > 0.05). Short- and long-term Cr supplementation does not influence functional capacity and calf muscle StO_2_ parameters in patients with symptomatic PAD.

## 1. Introduction

Walking impairment is the main clinical concern in patients with symptomatic peripheral artery disease (PAD). In these patients, walking impairment has been associated with increased barriers to physical activity practice [1], reduced physical activity levels [2], impaired cardiovascular profile [3,4], increased risk of limb loss, and a higher risk of cardiovascular disease and mortality [5,6]. Creatine (Cr) is a natural bioenergetic compound, which is converted to creatine phosphate or phosphocreatine and stored in muscle, where it is used for energy production [7]. Previous studies showed that a short period of oral Cr supplementation increases the amount of Cr available in the muscle by as much as 20% [8] and improves muscle performance in athletes [9] and healthy individuals [10]. In addition, Cr supplementation promotes improvements in handgrip strength and reduces lower-limb muscle fatigue [11,12]. In addition, long-term Cr supplementation increased muscle glycogen content [13,14], leading to an improvement in walking ability in clinical populations [15]. Therefore, as Cr has the ability to increase the muscle content of both phosphocreatine and glycogen, Cr supplementation could improve walking tolerance in patients with PAD, especially after the onset of claudication pain when anaerobic metabolism becomes predominant. Furthermore, a recent review [16] demonstrated the potential effects of Cr supplementation on vascular function and the lack of randomized controlled trials on this topic. Thus, the aim of this study was to analyze the effects of oral Cr supplementation on functional capacity and calf muscle oxygen saturation (StO_2_) in patients with symptomatic PAD.

## 2. Materials and Methods

### 2.1. Experimental Design

This was a randomized control pilot trial with a pre-test and post-test design. A 7 day, double-blind, placebo-controlled study was conducted from December 2016 to October 2017 in São Paulo, Brazil (registered at clinicaltrials.gov as NCT02993874). This manuscript is reported according to the CONSORT guidelines [17].

Patients were randomly assigned to the experiment in a 1:1 ratio, to blocks of 4–6, considering sex and total walking distance, to receive either placebo or creatine monohydrate supplementation according to a computer-generated treatment sequence in a double-blind design. The randomization process was carried out by a researcher who was not involved in the project. The primary outcome was total walking distance, as measured by the 6 min walk test (6MWT). Secondary outcomes were upper-limb strength (handgrip strength test), lower-limb strength (sit-to-stand test), and calf muscle StO_2_ assessed by near-infrared spectroscopy (NIRS). Participants were assessed at baseline (pre intervention), after 1 week of supplementation (loading), and after 8 weeks (maintenance). Throughout the protocol, both groups received clinical recommendations for patients with PAD [18].

### 2.2. Participant Recruitment and Screening

Patients were recruited from a tertiary vascular center in Sao Paulo, Brazil. The sample consisted of 29 patients of both sexes with PAD and symptoms of intermittent claudication. The inclusion criteria were the presence of intermittent claudication symptoms during the 6MWT, ankle brachial index <0.90 in one or both lower limbs, and the absence of chronic renal insufficiency (creatinine clearance <30 mL/min). Patients were excluded if they presented any side effects caused by Cr supplementation (i.e., gastric discomfort or diarrhea) or did not comply with the supplementation procedures. This study was approved by the Ethics Committee of the Institutions (process 62601416.7.0000.0071). All patients gave informed consent prior to participation.

### 2.3. Clinical Characteristics

Demographic information, height, weight, smoking history, and comorbid conditions (hypertension, dyslipidemia, diabetes, and coronary artery disease) were obtained through medical history and physical examination. Body mass (kg) and stature (m) were measured (Welmy, São Paulo, Brazil), and body mass index was calculated. The ankle brachial index was calculated as the ratio between ankle systolic and brachial systolic blood pressure as previously described [19]. A trained researcher performed all measurements.

### 2.4. Creatine Supplementation Protocol and Blinding Procedure

Patients received plain packages containing placebo (PLA) (dextrose) (Probiotica, Sao Paulo, Brazil) or creatine monohydrate (Cr) supplementation (Creapure, AlzChem Trostberg GmbH, Germany), 20 g/day for 1 week divided into four equal doses (loading phase), followed by single daily doses of 5 g for the next 7 weeks (maintenance phase). During the first 7 days (loading phase), supplements were presented in four packages and patients were instructed to ingest the packages at breakfast, lunch, dinner, and before bedtime. During the maintenance phase, patients consumed the supplement as a single dose with their lunch. The supplement packages were coded so that neither the investigators nor the participants were aware of the contents until completion of the analyses. Quality control and purity of the Cr were guaranteed by the manufacturer. The supplements were provided by a staff member of our research team who did not participate in acquisition, analyses, or interpretation of the data. Adherence to the supplementation was determined in a subsample of nine patients through plasma creatine levels, using high-performance liquid chromatography (FL SPD-20A Shimadzu^®^, Kyoto, Japan), as previously described [20].

Patients in both groups received the recommendation to increase their physical activity levels as recommended by vascular disease guidelines [18,19]. Physical activity was monitored using a previously validated pedometer (polar A300, Finland) [21], measured throughout the 8 weeks of supplementation. Patients were instructed to wear the monitor while awake and to remove it before bed.

### 2.5. Primary Outcome—6 Min Walk Test (6MWT)

Patients performed a 6MWT supervised by a trained kinesiologist. Two cones were placed 30 m apart in a marked corridor as previously described [22]. Patients were instructed to walk as many laps around the cones as possible and to report when the claudication symptom occurred. It is worth mentioning that none of the patients stopped during the test. The kinesiologist recorded the initial claudication distance (ICD) and total walking distance (TWD) to evaluate the walking capacity. In PAD patients, the 6MWT presents high reliability, with the intraclass correlation coefficient ranging between 0.94 and 1.00 and the coefficient of variation ranging between 0.4% and 1.6% [22].

### 2.6. Secondary Outcomes

#### Calf Muscle Oxygen Saturation (Calf Muscle StO_2_)

**Figure 1:**
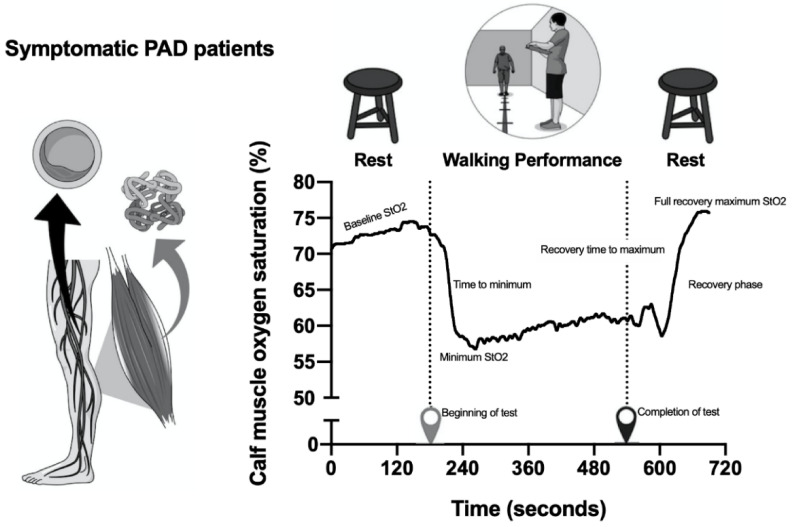
Calf muscle oxygen saturation (StO_2_) parameters obtained before, during, and after the 6 min walk test (6MWT) test in symptomatic peripheral artery disease (PAD).

Calf muscle StO_2_ was assessed during the 6MWT using near-infrared spectroscopy (PortaMon, Artinis Medical Systems) through a sensor attached on the leg with the lowest ankle brachial index. The sensor was attached to the skin on the medial portion of the gastrocnemius muscle, and several calf muscle StO_2_ parameters were obtained before, during, and after the 6MWT (Figure 1). Before the test, a baseline measure of calf muscle StO_2_ was obtained at rest (sitting position) for 3 min to allow stabilization of the values (baseline StO_2_). During exercise, the minimum calf muscle StO_2_ value (minimum StO_2_) was obtained, as the time taken to reach the minimum value (time to minimum) and the absolute drop in calf muscle StO_2_ from rest (baseline StO_2_) to the minimum exercise value (minimum StO_2_). The end of the 6MWT was recorded (completion of test), as well as the maximal calf StO_2_ value during recovery (full recovery maximum StO_2_) in the sitting position. The recovery times for calf muscle StO_2_ to reach the full resting value (recovery phase time) and the maximum calf muscle StO_2_ value (recovery time to maximum) were calculated [23].

### 2.7. Handgrip Strength Test

Handgrip strength was measured using a digital hand dynamometer (Model HE101, WCT fitness). The measurement was performed with the participants in a sitting position, with the shoulder adducted in a neutral position and without rotation. The elbow joints were positioned at 90° flexion, with the forearm and wrist in a neutral position. Measurements were performed three times on both arms and the highest value was considered.

### 2.8. Sit-to-Stand Test

Participants were asked to start by sitting with their feet on the floor and their upper limbs bent over the chest, and then to stand up and sit down again without using their arms. Participants repeated the action five times as quickly as possible, and the time required to complete the five repetitions was recorded [24].

### 2.9. Statistical Analysis

The sample power was calculated using the G* Power software 3.19 statistics program. Thus, considering that the current sample included 29 patients (PLA, *n* = 15; Cr, *n* = 14) with an effect size of 1.91 and alpha error of 0.05, the sample power was 0.80. The normality of the data was verified using the Shapiro–Wilk test. The comparison between the general characteristics of the sample was performed using the *t*-test for independent samples. The categorical data were compared using the chi-square test. The baseline values were assessed using the Mann–Whitney U test. To verify the effect of supplementation, the generalized estimating equation model for repeated measures was performed. The level of significance was *p* < 0.05.

## 3. Results

The characteristics of the groups are presented in Table 1. Groups were similar at baseline in all clinical characteristics (*p* > 0.05).

**Figure 2:**
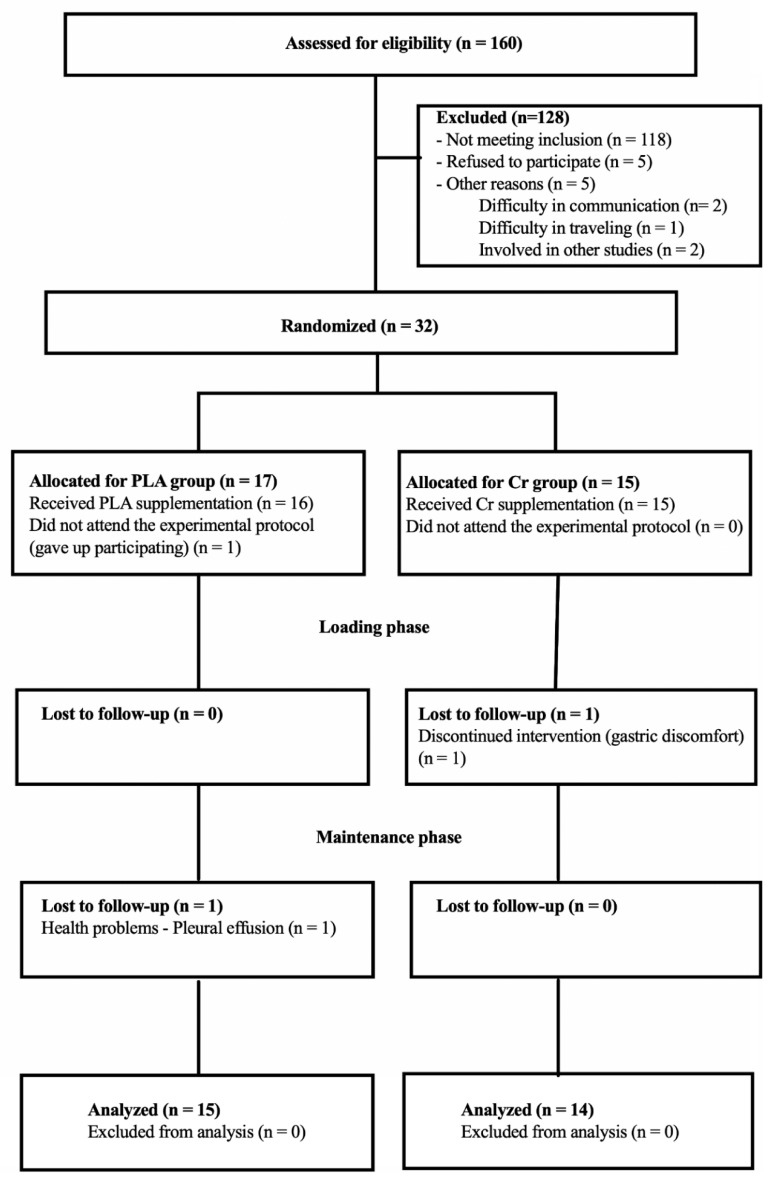
Study flow.

Initially, 160 patients were interviewed for eligibility; of these, 118 did not meet the inclusion criteria, five refused to participate, and five were not included for other reasons such as not answering phone calls (two subjects), difficulty in traveling because they lived far away (one patient), and being involved in another study (two patients). Thus, 32 patients started the study, with 17 allocated to the placebo group and 15 to the Cr group. Before the beginning of the protocol, one patient was excluded for not attending the evaluation (placebo; *n* = 1). During the loading period, one patient receiving Cr was excluded due to gastric discomfort (*n* = 1). During the maintenance supplementation period, one patient was excluded for presenting pleural perfusion (placebo group). Twenty-nine patients completed the study (PLA, *n* = 15; Cr, *n* = 14) (Figure 2).

Patients taking Cr presented higher plasma creatine levels than the placebo group (PLA: pre 21.5 ± 38.9 μmol/L vs. post 30.7 ± 39.8 μmol/L; Cr: pre 32.1 ± 61.4 μmol/L vs. post 163.2 ± 42.65 μmol/L; time × intervention effect, *p* = 0.042).

**Figure 3:**
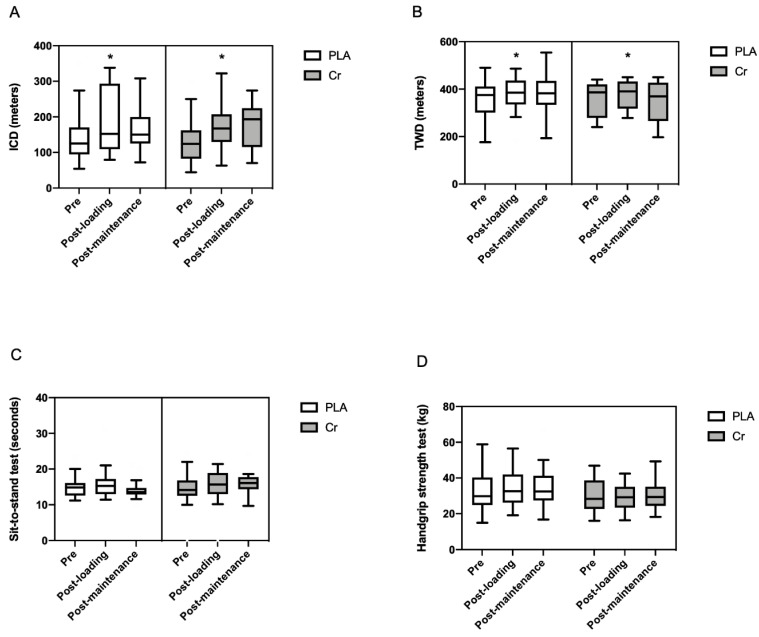
Functional capacity parameters obtained during the 6MWT test, handgrip strength test, and sit-to-stand test before (pre) and after the loading and maintenance supplementation period in symptomatic peripheral arterial disease (*n* = 29; PLA = 15 and Cr = 14). Data are expressed as the median and interquartile range. * Significant difference pre vs. post loading supplementation period (*p* < 0.05). (**A**) ICD = initial claudication distance; (**B**) TWD = total walking distance; (**C**) sit-to-stand test; (**D**) handgrip strength test. PLA—placebo group; Cr—creatine group.

No significant interactions were found for ICD (Figure 3A) (PLA: pre 124 ± 72 m vs. post loading 150 ± 199 m vs. post maintenance 145 ± 90 m; Cr: pre 124 ± 73 m vs. post loading 168 ± 90 m vs. post maintenance 193 ± 110 m; time × intervention effect, *p* = 0.532) and TWD (Figure 3B) (PLA: pre 389 ± 123 m vs. post loading 413 ± 131 m vs. post maintenance 382 ± 99 m; Cr: pre 373 ± 149 m vs. post loading 390 ± 115 m vs. post maintenance 369 ± 115 m; time × intervention effect, *p* = 0.170). However, we observed a significant increase in ICD from pre to post loading in both groups (time effect, *p* = 0.009).

For physical activity levels (number of steps), no differences were found at pre vs. post supplementation (PLA: pre 457 ± 652 steps/day vs. post maintenance 465 ± 602 steps/day; Cr: pre 584 ± 360 steps/day vs. post maintenance 599 ± 314 steps/day; time effect = 0.258; time × intervention effect, *p* = 0.357).

There were no changes in the sit-to-stand test (Figure 3C) (PLA: pre 14.8 ± 2.9 s vs. post loading 15.1 ± 3.2 s vs. post maintenance 13.6 ± 1.5 s; Cr: pre 14.1 ± 5.1 s vs. post loading 15.6 ± 5.9 s vs. post maintenance 16.1 ± 2.2 s; time × intervention effect, *p* = 0.400) or handgrip strength test (Figure 3D) (PLA: 32.5 ± 16.0 kgf vs. post loading 34.3 ± 15.4 kgf vs. post maintenance 32.4 ± 13.7 kgf; Cr: pre 28.3 ± 15.2 kgf vs. post loading 29.2 ± 11.7 kgf vs. post maintenance 29.3 ± 10.2 kgf; time × intervention effect, *p* = 0.251).

The effects of supplementation on calf muscle StO_2_ parameters are presented in Table 2. No significant time × intervention interaction was found for any parameter (*p* > 0.05).

## 4. Discussion

The main findings of this pilot study were that short- and long-term Cr supplementation did not improve functional capacity and calf muscle StO_2_ in symptomatic PAD patients.

Previous studies demonstrated that short- and long-term Cr supplementation increases physical performance in healthy subjects [10,25,26] and in patients with chronic diseases [15]. In contrast, our findings demonstrate no effects of Cr supplementation on 6MWT in symptomatic PAD patients. A potential explanation is that Cr rapidly increases the energy stores in type II fibers, and pain during walking in symptomatic PAD occurs mainly in calf muscles, predominantly composed of type I fibers [27]. Another factor may be related to the combination of Cr supplementation with other nutritional supplements. Benedetto et al. [15] analyzed the effects of 8 weeks of Cr supplementation combined with Coenzyme Q10 on walking capacity (6MWT) in patients with chronic obstructive pulmonary disease, and they demonstrated a significant increase of 51 m in the Cr supplementation group compared to the placebo group. In fact, Coenzyme Q10 is a compound that acts on the electron transport chain participating in cell respiration, helping to generate energy in the form of ATP [28]. As we only used Cr supplementation without the combination of other nutritional supplements, it is possible that the effects of Cr in the muscle were attenuated. Thus, future studies are necessary to investigate whether a combination of different nutritional supplements can improve physical performance in symptomatic PAD patients.

We also analyzed the microcirculation during the 6MWT using the calf muscle StO_2_ technique. Cr acts on microcirculation due to the increased activity of sensory nerves and epoxygenase metabolites, particularly epoxyethane diacetic acid, related to the endothelium-derived hyperpolarization factor [29]. Previous studies showed that Cr supplementation improved calf muscle StO_2_ in healthy young people [30] and vegan individuals [31]. However, in our study, Cr supplementation did not change any calf muscle StO_2_ parameters, which agrees with the results of the 6MWT. Thus, these results support that only Cr supplementation would not be enough to promote changes in microcirculation and physical performance in symptomatic PAD patients.

In the present study, Cr supplementation did not change other functional capacity tests, such as the sit-to-stand test and handgrip strength. The positive effects of Cr supplementation in tests involving muscle strength, fatigue, and endurance are controversial. For example, while Rawson et al. [11] demonstrated that 4 weeks of Cr supplementation reduced muscle fatigue assessed with isokinetic dynamometry in healthy older people, Lobo et al. [32] did not find any adjuvant effect after 1 year of Cr supplementation on lower-limb strength (sit-and-stand test) in postmenopausal older women. Taken together, these results suggest that the effects of Cr supplementation on muscle function are influenced by sample characteristics and methods to assess muscle function.

Our study presents some limitations. Although we analyzed the amount of creatine in the blood [33], we did not measure the content of creatine in the muscle and, thus, the impact of supplementation on the calf muscle was not determined. Our findings are restricted to the time of supplementation and the dosage of creatine used in this study. Furthermore, a limitation is the small sample size, and more studies are necessary with larger sample. Other tests to assess muscle function could be used, such as isokinetic dynamometry and a treadmill. However, we included tests to assess the strength in upper and lower limbs and the 6MWT, which represent greater clinical relevance in these patients [34,35,36]. Lastly, we only analyzed the effects of Cr supplementation for 8 weeks, and more studies with longer periods of supplementation are necessary.

## 5. Conclusions

Our results demonstrated that a short period (loading for 1 week) and a long period (maintenance for 8 weeks) of Cr supplementation do not increase functional capacity (walking distance, and upper- and lower-limb muscle strength) and calf muscle StO_2_ in patients with symptomatic PAD. This pilot study is applicable to the development of new Cr supplementation strategies to improve the performance and health outcomes in PAD population.

## Figures and Tables

**Table 1:** Characteristics of participants at baseline (*n* = 29).

	PLA (*n* = 15)	Cr (*n* = 14)	*p*-Value
Women (%) ^a^	54	46	0.56
Age (years) ^a^	64 ± 8	64 ± 10	0.54
Weight (kg) ^a^	77 ± 10	68 ± 17	0.18
Height (m) ^a^	1.64 ± 0.09	1.60 ± 0.06	0.21
Body mass index (kg/m^2^) ^a^	28.7 ± 3.1	26.7 ± 6.5	0.43
Ankle-brachial index (mmHg) ^a^	0.50 ± 0.13	0.51 ± 0.16	1.00
Initial claudication distance (m) ^a^	143 ± 84	143 ± 65	0.88
Total walking distance (m) ^a^	371 ± 81	344 ± 82	0.65
Comorbidities (%)			
Hypertension ^b^	86.7	78.6	0.67
Diabetes ^b^	60.0	50.0	0.43
Dyslipidemia ^b^	6.7	7.1	0.74
Current smoking ^b^	78.6	78.6	0.68
Coronary artery disease ^b^	46.7	28.6	0.26

Data are presented as the mean and standard deviation for numerical variables and frequency for categorical variables. ^a^ *t*-Test for independent samples. ^b^ Chi-square test. PLA—placebo group; Cr—creatine group.

**Table 2:** Calf muscle StO_2_ data before (pre) and after the short period (post loading) and long period (post maintenance) of supplementation (*n* = 29).

	PLA (*n* = 15)	Cr (*n* = 14)	*p*-Value Interaction
**Baseline StO_2_ (%)**			
Pre	68.8 ± 3.7	70.0 ± 4.6	0.082
Post loading	68.2 ± 2.8	70.5 ± 6.1	
Post maintenance	71.3 ± 5.7	71.1 ± 4.8	
**Minimum StO_2_ (%)**			
Pre	54.2 ± 10.0	61.6 ± 13.0	0.191
Post loading	52.9 ± 9.3	61.7 ± 14.8	
Post maintenance	54.2 ± 7.7	62.8 ± 12.3	
**Time to minimum StO_2_ (s)**			
Pre	52.5 ± 34.3	56.5 ± 46.1	0.833
Post loading	46.8 ± 58.6	44.4 ± 26.2	
Post maintenance	50.1 ± 88.3	58.4 ± 43.5	
**Completion of test (%)**			
Pre	12.2 ± 8.5	7.2 ± 9.7	0.691
Post loading	12.1 ± 8.2	9.5 ± 7.1	
Post maintenance	9.2 ± 10.5	7.6 ± 10.4	
**Recovery time to maximum StO_2_ (s)**			
Pre	158.9 ± 43.1	160.1 ± 175.1	0.723
Post loading	146.4 ± 75.1	177.1 ± 202.9	
Post maintenance	160.9 ± 119.1	181.9 ±125.8	
**Recovery phase time StO_2_ (s)**			
Pre	126.7 ± 112.4	132.9 ± 194.2	0.827
Post loading	93.0 ± 77.7	121.1 ± 248.3	
Post maintenance	87.1 ± 137.5	130.3 ± 103.7	
**Full recovery maximum StO_2_ (%)**			
Pre	69.5 ± 15.8	72.6 ± 12.4	0.312
Post loading	68.6 ± 9.7	72.3 ± 14.5	
Post maintenance	74.5 ± 15.8	73.1 ± 15.8	

Data are expressed as the median and interquartile range. PLA—placebo group; Cr—creatine group.
